# Mouse Phenome Database: a data repository and analysis suite for curated primary mouse phenotype data

**DOI:** 10.1093/nar/gkz1032

**Published:** 2019-11-07

**Authors:** Molly A Bogue, Vivek M Philip, David O Walton, Stephen C Grubb, Matthew H Dunn, Georgi Kolishovski, Jake Emerson, Gaurab Mukherjee, Timothy Stearns, Hao He, Vinita Sinha, Beena Kadakkuzha, Govindarajan Kunde-Ramamoorthy, Elissa J Chesler

**Affiliations:** The Jackson Laboratory, Bar Harbor, Maine, ME 04609, USA

## Abstract

The Mouse Phenome Database (MPD; https://phenome.jax.org) is a widely accessed and highly functional data repository housing primary phenotype data for the laboratory mouse accessible via APIs and providing tools to analyze and visualize those data. Data come from investigators around the world and represent a broad scope of phenotyping endpoints and disease-related traits in naïve mice and those exposed to drugs, environmental agents or other treatments. MPD houses rigorously curated per-animal data with detailed protocols. Public ontologies and controlled vocabularies are used for annotation. In addition to phenotype tools, genetic analysis tools enable users to integrate and interpret genome–phenome relations across the database. Strain types and populations include inbred, recombinant inbred, F1 hybrid, transgenic, targeted mutants, chromosome substitution, Collaborative Cross, Diversity Outbred and other mapping populations. Our new analysis tools allow users to apply selected data in an integrated fashion to address problems in trait associations, reproducibility, polygenic syndrome model selection and multi-trait modeling. As we refine these tools and approaches, we will continue to provide users a means to identify consistent, quality studies that have high translational relevance.

## INTRODUCTION

The Mouse Phenome Database (MPD) provides a unique repository for the integration and aggregation of mouse phenotype data with fully documented protocols, annotation to rigorously developed ontologies (1–3), and modular interoperation with a suite of innovative tools for integrative analysis. Users are able to perform analyses for research questions based on results obtained from the MPD data archive. MPD has an interactive environment with dynamic visualizations of measures, enabling investigators to discover relationships among genes, variants and phenotypes through primary data, ontology annotations and protocols, resulting in a better understanding of the clinical manifestations they aim to model.

Curation of measures and other entities with widely used ontology terms is important for integration with multi-species platforms such as the Monarch Initiative (4) and GeneNetwork (5). By structuring mouse phenotyping studies, annotating them to controlled vocabularies and developing integrative tools that rely on the unique value of these data, MPD facilitates access and reuse of heterogeneous primary phenotype data, enabling cross-population and cross-species comparisons and assessment of trait relevance to human studies.

MPD houses phenotype and/or genotype data for over 2000 strains and populations. Data come from any verifiable mouse strain or population and are registered with proper strain nomenclature. Animals can be representatives of strains with defined genetic backgrounds or members of populations comprised of individuals with unique recombinant genomes. Strain types include inbred, recombinant inbred, F1 hybrid, transgenic, targeted mutant, chromosome substitution and Collaborative Cross (6,7). Populations include offspring from inbred strain F2 crosses, backcrosses and other experimental crosses found in the QTL Archive, Diversity Outbred mice (7,8), and other heterogeneous stocks such as the UM-HET3 mice as used by the National Institute on Aging Interventions Testing Program (9).

By placing these genetic data together into a single data resource, many applications are readily enabled, including:

1. Comparison of baseline and treatment data
2. Using multiple measures to choose optimal strains for:
3. Modeling human diseases and conditions
4. Creating engineered mutations on sensitive genetic backgrounds (e.g. CRISPR/cas9)
5. Mapping studies
6. Physiological studies
7. Drug studies
8. Elucidating shared genetics for correlated traits
9. Discovering convergent genotype-phenotype relationships across populations
10. Studying sex differences and sex-by-genotype interactions
11. Assessing replicability of traits across experiments and laboratories
12. Providing validated protocols and reference data collected under those protocols

Here, we present several advances and new features since our last NAR update.

## NEW FEATURES AND IMPROVEMENTS

Since our last NAR report, we have made the following major improvements to enhance capabilities and user experience:

1. New tools have been deployed for univariate and multivariate analysis.
2. Data visualizations have been upgraded and are highly interactive.
3. Results from multiple tools are presented in a dashboard format.
4. A new ontology browser has been deployed.
5. Documented APIs are available for tool prototyping, offline analysis and use by other resources.
6. An expanded data dictionary has been employed, including metadata for variable types and other experimental design characteristics used in tool selection.

### Phenotype data analysis

Our new tool set is built using R statistical software, Python and D3 visualizations. These rigorous analyses complement our existing tool set. We have re-engineered several tools, including Find Strains by Criteria Fit, Ratios and Differences, Scatterplots and Correlations, Side-by-Side View and Curves Comparison View. Find Strains by Criteria Fit (not shown) is a powerful tool based on *Z*-scores to identify strains exhibiting particular phenotypic profiles. Users select measures of interest (up to 20 measures can be examined simultaneously) and apply criterion for each measure (high-end outlier, low-end outlier or average). Strains are then listed in a dynamic table in order of best fit. This tool is complemented by a multivariate outlier detection statistical tool (see below), which does not specify the magnitude and direction of contrasts, but allows detection of statistical outliers based on a method that scales to high-dimensional datasets. The Ratios and Differences tool plots ratios or differences for two selected measures. This is particularly good for determining the change in phenotypes under differing conditions, e.g., change in body weight after 8 weeks of high-fat diet. It is complemented by factorial mixed model analysis of variance for estimating statistical interactions among predictive cofactors and covariates. Time series and other models are being included in future releases.

**Figure 1.**
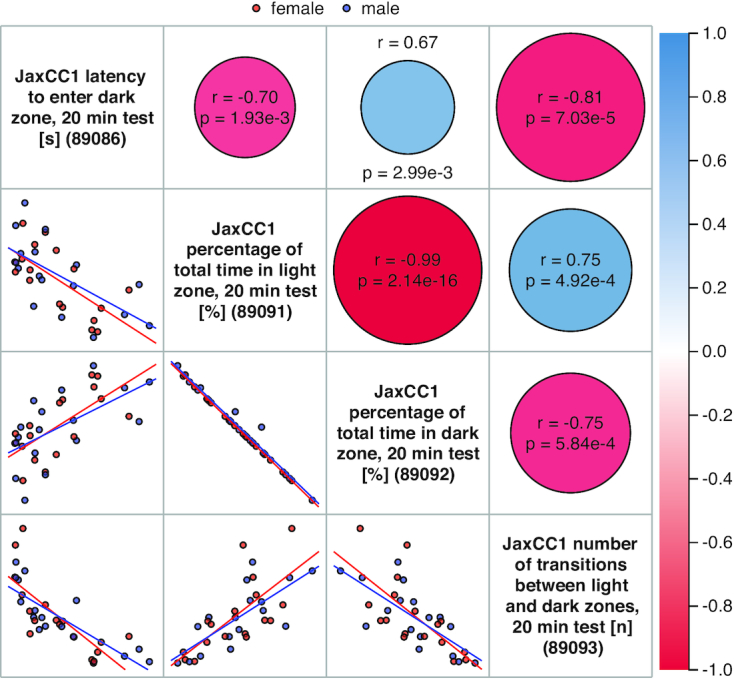
Scatterplot and Correlations tool. Below the diagonal, female (red) and male (blue) data points are seen along with regression lines in thumbnail scatterplots. Above the diagonal in cells corresponding to scatterplots, positive correlations are in blue and negative correlations are in red. Pearson correlation coefficients (*r*) and *P*-values are indicated for each associated scatterplot. Clicking on the measure name along the diagonal will take users to a plot of that measure with summary statistics (not shown). Clicking on any other cell above or below the diagonal will take users to a larger, more detailed scatterplot with various viewing options (not shown).

The Scatterplots and Correlations tool (Figure 1) displays thumbnail scatterplots in a grid (below the diagonal) that also contains visual aids (above the diagonal) that are coded to help users quickly identify significant correlations (the more intense the color, the higher the absolute value of the correlation coefficient) and *P*-values (the larger the circle, the smaller the *P*-value). The default grid uses least squares means, but regular means can also be analyzed/visualized. There is also an option to view individual animal data points as well as options for adjusting the size of the grid, hiding/showing the regression line, plotting Pearson or Spearman and displaying unadjusted or adjusted *P*-values. There are various download options. Clicking on any cell will take the user to a larger scatterplot (not shown) where there is detailed data and more viewing options, e.g., opt to see strain names, to see male only or female only, to see error bars and to flip axes. Clicking on a strain name will take a user to a strain detail page (not shown) where all MPD data on that strain is indexed through links and where there is a search box to get to data of interest more quickly. There are also link-outs to vendor websites (if available) and to Mouse Genome Informatics (MGI) strain detail pages (10). The Scatterplots and Correlations tool is useful for elucidating relationships among traits as shown in Figure 1 for Collaborative Cross strains.

**Figure 2.**
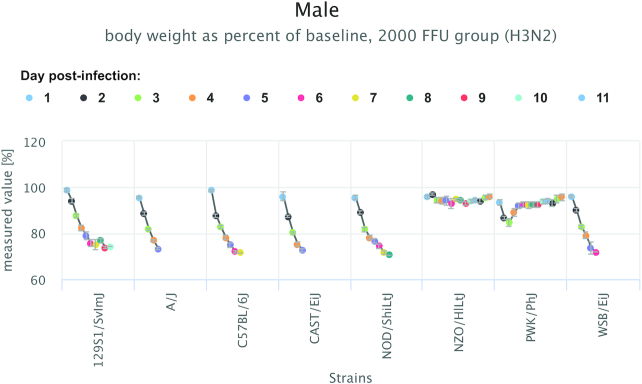
Side-by-side strain comparison view of data in a series. Strain-specific phenotypic profiles and trends in the data are made obvious in this plot type, where a repeated measure is plotted over time.

Strain data from a series of measures is plotted in the Side-by-Side View (Figure 2), as opposed to a parallel coordinates plot where all strains are plotted in an overlapping manner in the same space (not shown). The Side-by-Side view is useful for identifying strains that show extreme phenotypes across a series of measures. Means are plotted by default but users could opt to see *Z*-scores plotted instead. Data can be plotted as points (as shown in Figure 2) or as bars (not shown). Females and males are plotted separately. Error bars can be hidden and the dimensions of the plot can be altered. The plot itself is interactive where users can mouse-over data points and generate a pop-up box containing mean, standard deviation and standard error. There are various download options.

**Figure 3.**
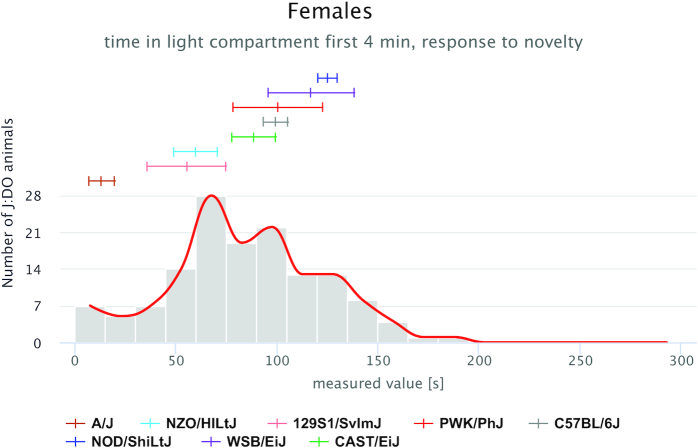
Diversity Outbred mice with eight founder inbred strains. The histogram shows the distribution of DO mice for this measure, while means and standard errors of the founder strains are shown above the histogram. Strains are color-coded based on community standards (see plot legend).

In addition to these new views, we have implemented specialized plots for mapping populations, including Diversity Outbred (DO) data (Figure 3). DO data are plotted as a histogram of individual animal observations, and founder strains means and standard errors are plotted above the histogram (color-coded). Females and males are plotted separately. The bin number can be altered in order to smooth the histogram curve. DO data can also be viewed in a box-and-whisker plot with individual DO animals plotted (not shown).

**Figure 4.**
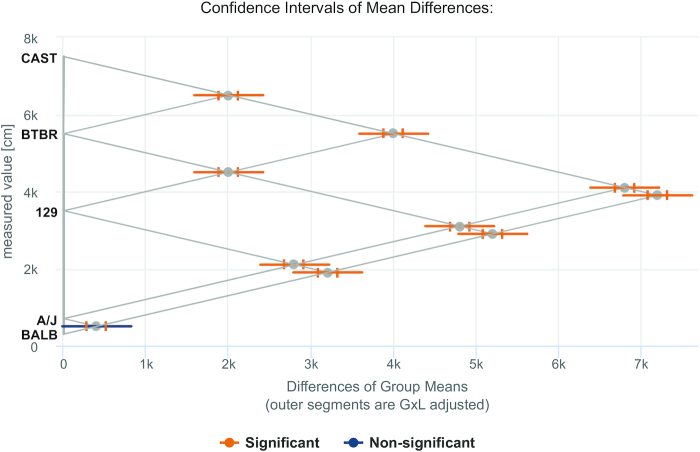
GxL Replicability Adjuster Tool: Comparison Plot. Differences of strain means are plotted for pairwise comparisons (see abbreviated strain name along the *y*-axis). Inner segments are unadjusted confidence intervals while the outer segments indicate the GxL adjusted confidence intervals. Orange indicates significant results (*P*≤ 0.05) while blue indicates non-significant.

MPD has consistently provided rigorous curation of mouse experimental data to help provide insight into issues associated with reproducibility and replicability (11,12). We have implemented the Genotype-by-Laboratory (GxL) Replicability Adjuster Analysis tool designed by Yoav Benjamini *et al.* at Tel Aviv University based on their Random Laboratory Model (13,14). This tool takes a user’s input phenotype data (from a single laboratory), compares two or more genotypes (strains), and adjusts the *P*-values and confidence intervals to indicate the likelihood of replicability in another lab. Users must first select three or more MPD measures of interest that were collected under similar protocols and conditions as their own data. The MPD measures are the reference measures from which the replicability adjuster estimate is computed. Users can upload their data in one of two ways: comma-delimited file with individual animal observations or manually enter summary statistics for each of the strains (mean, standard deviation and number of mice). The application runs GxL-adjusted *t*-tests for the pairwise differences between groups. It outputs the GxL-adjusted *P*-values and confidence intervals of the pairwise differences, alongside the standard *P*-values and confidence intervals. Results are tabular (not shown) and graphical (boxplot (not shown), comparison plot (Figure 4)). To assist users in identifying relevant data for their reference measures, we are structuring relevant protocol information for a comparison view of measure meta-content.

**Figure 5.**
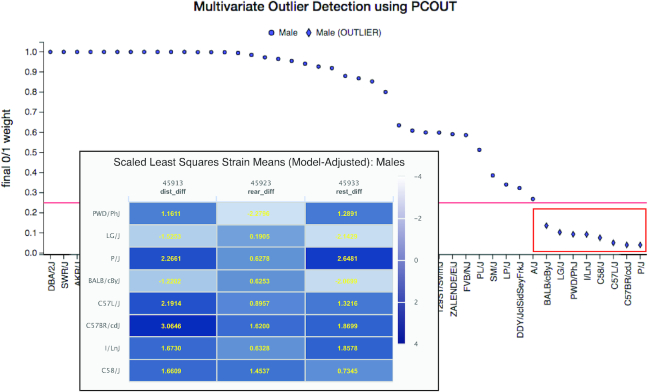
Polygenic Syndromic Model Selection Tool. User-selected measures are analyzed simultaneously for multivariate outliers. Results are presented in a 2D plot where outliers are indicated below a red cut-off line. The tool allows the user to select strains of interest (red box) whereby a visualization of results appears below the plot (see inset). The table is color-coded so that users can quickly identify strains of interest based on their phenotypic profiles across the measures.

We are developing tools for multivariate phenotype analysis. A multivariate outlier detection tool has recently been deployed for multivariate trait modeling (Figure 5). This is particularly useful when applied to data from populations like the Collaborative Cross panel for identifying new, complex, polygenic mouse models of human biology and disease. Users select their traits of interest (maximum 15) and issue the query. The results of the analysis are visualized in a 2D plot where outlier strains are plotted below a red cut-off line. Users can brush over (click and select) outlier strains to generate a color-coded table of results, where they can choose strains for their research applications based on particular phenotypic profiles.

We have deployed a new ontology browser that is accessed when searching on an ontology term in the main search box at the top of any MPD page. A link is provided that takes users to a webpage of that term with parent term(s) indicated and a link to the root of that particular ontology. From here, users can opt to search ontologies where all terms are examined and search results return all instances of ontology terms containing the search word(s). Clicking on an ontology term from here then takes users to a results table of measures that have been annotated to that particular ontology term. Users can then apply eligible analysis tools to this ontology-term-specific set of measures.

### Genotype data

We have incorporated a new genotype dataset since our last NAR paper from the Eleazar Eskin lab at UCLA (132K SNPs across ∼250 strains; we call this the UCLA1 dataset). This dataset has been added to the existing collection of data from inbred, recombinant inbred, Collaborative Cross and chromosome substitution strains. We are planning to integrate all SNP genotypes into a single imputed SNP resource. In the meantime, users can access the SNP-retrieval utility through the ‘Genotype’ tab on the homepage (not shown) or through this quick link: https://phenome.jax.org/genotypes. We provide a SNP query form where users can enter gene symbols (MGI), coordinates (in basepairs or Mbp), or rs numbers either singly or in various combinations (up to 50 items may be listed). Users can select desired flanking sequence, and results can be filtered on dbSNP functional annotations. Users can query entire chromosomes or even the entire genome.

### Phenotype–Genotype

**Figure 6.**
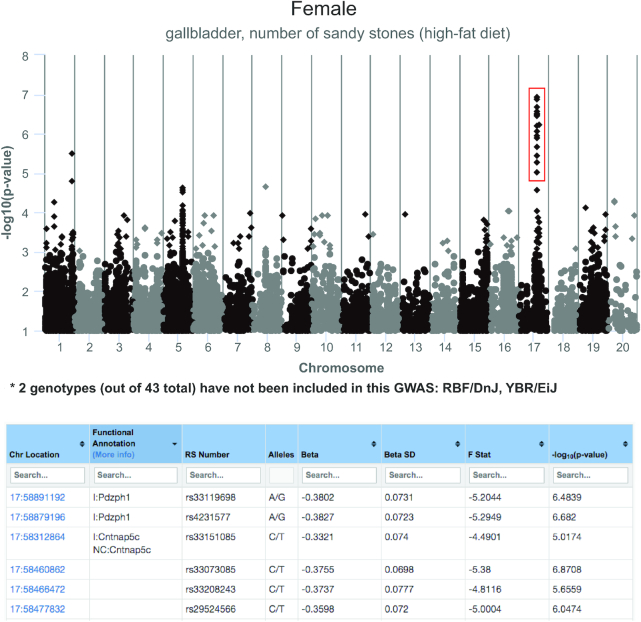
GWAS tool based on PyLMM. A Manhattan plot of the results is provided (top panel). Data points (SNPs) can be selected (red box) and viewed in a searchable and sortable table (bottom panel). Chromosome, location, dbSNP functional annotation, rs (reference SNP accession) number, alleles and PyLMM output data are available in the table. Users can gather genes or rs numbers in a list to use as input for external research applications.

We have implemented a GWAS tool based on PyLMM (Eleazar Eskin lab, UCLA) which is a python-based linear mixed model tool that accounts for population structure inherent in GWAS studies (Figure 6). Our current implementation of this tool uses genotype data from the UCLA1 dataset mentioned above. In the near future, we will employ LocusZoom (15) to display users’ regions of interest in detail. In the meantime, users can brush over (click and select) SNPs in the Manhattan plot to generate a table of SNPs with location, genes with dbSNP functional annotations, rs numbers and PyLMM output results (as shown in Figure 6).

### Future directions

Modular tools for multivariate phenotype analysis and trait meta-analysis are being integrated in our tool set. The redesigned architecture of the MPD is amenable to the collaborative contribution of additional modules, and the APIs further allow independent analysis tools to operate off the data in the MPD repository without tightly coupled integration.

We are developing a Data Intake and Annotation Interface in which a registered user can define a new project and upload their experimental phenotype data. They will be given an easy-to-use interface, similar to a spreadsheet view, to modify a pre-populated data dictionary describing the variables with metadata. This information includes variable types, ontology annotations, distributional properties and study design information for use in modeling via identification of factors, covariates, and series measures. The data descriptions will be used to specify tools and displays that are valid for a given dataset. MPD will serve as the primary platform for phenotypic data curation for systems genetics resources such as GeneNetwork (5) and Diversity Outbred and Collaborative Cross projects (16). Curatorial review of the submissions will be performed by MPD staff before making new data part of the public repository.

## ACCESSING AND SUBMITTING DATA

### Bulk and programmatic access

Bulk data downloads are available at https://phenome.jax.org/downloads in csv format. Excel can be used in local environments. A set of public API endpoints is available for programmatic access to specific phenotype data (individual animal data or strain means), metadata and analytics results (all returned in JSON or csv format). For more information see https://phenome.jax.org/about/api.

### Data submission

Most of the data in MPD are directly contributed by investigators worldwide. Other data are acquired through public resources. Data submissions can have various study designs including baseline characterizations, diet studies, drug and alcohol studies and other treatment versus control studies, aging and longevity studies, challenge/pathogen studies, and more. A short synopsis of the project and study design are usually included. We accept data from all strain types and populations, ensuring annotation to current nomenclature. Besides a dataset, we require measurement descriptions and units. Free text meta-content is requested to help users quickly understand the study. In addition to the dataset and measurement descriptions, we require a detailed protocol and information about housing and testing environments, which is particularly important for the GxL Replicability Adjuster tool. Research resource identification numbers (RRIDs) (17) are strongly recommended to unambiguously identify reagents and resources used in the study. Funding sources and other acknowledgments can be added to the project. If available, a primary publication can be linked to the project. We encourage investigators to publish their findings in peer-reviewed journals. In many cases, submission to MPD satisfies journal mandates for public release of data.

As mentioned above, we are currently implementing an interface for data contributors so that they may directly assist in curating their own data and creating data dictionaries to their specifications. This added feature will streamline the submission process, making data publicly available more rapidly than currently possible. Until this feature is released, data contributors should follow our data submission guidelines found at https://phenome.jax.org/about/contributedata. Submissions and questions should be sent to phenome@jax.org.

## IMPLEMENTATION AND PUBLIC ACCESS

The MPD system is hosted on a suite of CentOS Linux virtual machines (VMs). MPD is served by Apache and utilizes NetScaler for firewall and SSL support. The public site is implemented using Python, Flask and PostgreSQL relational database, with a user interface implemented with Jinja2 templates, Bootstrap, JavaScript and JQuery. MPD is readily accessible and legible on tablet screens, and some content is reasonably accessed through phone screens. The system also includes an analysis server for all of the computations including long running preprocessing of new measures and real-time analysis for public analytical tools. The analytic services are hosted using a suite of Docker containers, including a RESTful API implemented with Python and Flask REST-Plus, and an R-based ‘mpdanalysis’ analytic package. All of the services are replicated in a dev/test environment to allow the MPD team to implement new functionality without impacting users.

The software development team uses Git and Bitbucket to manage code, Confluence for documentation, and JIRA for project management of our Agile process, including tracking of releases, sprints and issues. Public releases are every six 6 weeks with 2-week iteration cycles. Automated testing is done at the unit, regression and functional level, the latter being implemented in Groovy, using Geb and Spock. Unit testing is done with the Python built-in *unittest* module.

## CITING MPD

For a general citation of the MPD resource, researchers should cite this article and use RRID:SCR_003212. The following citation format is suggested when referring to MPD datasets: Investigator(s) name(s) [last name, first initial, middle initial]. Title of project. MPD:project symbol [such as Smith1]. Mouse Phenome Database web resource (RRID:SCR_003212), The Jackson Laboratory, Bar Harbor, Maine USA. https://phenome.jax.org [Cited (date)]. The content is solely the responsibility of the authors and does not necessarily represent the official view of the NIH.
